# Assessment of stress levels and reproductive condition in giant pandas: insights from hair, faecal and saliva samples

**DOI:** 10.1093/conphys/coae044

**Published:** 2024-07-03

**Authors:** Zheng Yan, Xiaoyan Liu, Haoqiu Liu, Haihong Xu, Yanhui Liu, Changqing Li, Bo Wang, Shengnan Cui, Ting Jia, Di Yang, Chenglin Zhang, Xuefeng Liu, Christina D Buesching, Dingzhen Liu

**Affiliations:** Key Laboratory for Biodiversity and Ecological Engineering of Ministry of Education, Department of Ecology, College of Life Sciences, Beijing Normal University, No.19, Xinjiekouwai Street, Haidian District, Beijing 100875, China; Key Laboratory for Biodiversity and Ecological Engineering of Ministry of Education, Department of Ecology, College of Life Sciences, Beijing Normal University, No.19, Xinjiekouwai Street, Haidian District, Beijing 100875, China; Key Laboratory for Biodiversity and Ecological Engineering of Ministry of Education, Department of Ecology, College of Life Sciences, Beijing Normal University, No.19, Xinjiekouwai Street, Haidian District, Beijing 100875, China; Beijing Key Laboratory of Captive Wildlife Technologies, Beijing Zoo, No.137, Xizhimenwai Street, Xicheng District, Beijing 100044, China; Beijing Key Laboratory of Captive Wildlife Technologies, Beijing Zoo, No.137, Xizhimenwai Street, Xicheng District, Beijing 100044, China; Beijing Key Laboratory of Captive Wildlife Technologies, Beijing Zoo, No.137, Xizhimenwai Street, Xicheng District, Beijing 100044, China; Beijing Key Laboratory of Captive Wildlife Technologies, Beijing Zoo, No.137, Xizhimenwai Street, Xicheng District, Beijing 100044, China; Beijing Key Laboratory of Captive Wildlife Technologies, Beijing Zoo, No.137, Xizhimenwai Street, Xicheng District, Beijing 100044, China; Beijing Key Laboratory of Captive Wildlife Technologies, Beijing Zoo, No.137, Xizhimenwai Street, Xicheng District, Beijing 100044, China; Beijing Key Laboratory of Captive Wildlife Technologies, Beijing Zoo, No.137, Xizhimenwai Street, Xicheng District, Beijing 100044, China; Beijing Key Laboratory of Captive Wildlife Technologies, Beijing Zoo, No.137, Xizhimenwai Street, Xicheng District, Beijing 100044, China; Beijing Key Laboratory of Captive Wildlife Technologies, Beijing Zoo, No.137, Xizhimenwai Street, Xicheng District, Beijing 100044, China; Department of Biology, Irving K. Barber Faculty of Sciences, The University of British Columbia, Okanagan, Kelowna, British Columbia V1V 1V7, Canada; Key Laboratory for Biodiversity and Ecological Engineering of Ministry of Education, Department of Ecology, College of Life Sciences, Beijing Normal University, No.19, Xinjiekouwai Street, Haidian District, Beijing 100875, China

**Keywords:** chronic stress, conservation, cortisol, estrogen, fur, giant panda, non-invasive hormone monitoring, progesterone, sex steroid, testosterone

## Abstract

Concerted conservation efforts have brought the giant panda (*Ailuropoda melanoleuca*) back from the brink of extinction, but pandas continue to face anthropogenic threats in the wild and breeding success in captivity remains low. Because stress can have detrimental impacts on reproduction, monitoring stress- and sex-steroid levels would help assess the effectiveness of conservation mitigation measures in panda populations as well as monitor the welfare and reproductive health of captive animals. In this proof-of-concept study, we used faecal sex steroid and cortisol concentrations (*n* = 867 samples collected from five males and five females at Beijing Zoo every 4 days over the course of 12 months) as a reference to investigate if testosterone, estradiol, progesterone and cortisol can be meaningfully measured in panda hair (*n* = 10) using radio-immuno-assays. Additionally, we calculated the ratio of testosterone to cortisol (T:C ratio) for each male, which can provide a biomarker of stress and physical performance. Our findings revealed distinct monthly variations in faecal sex-steroid and cortisol concentrations, reflecting reproductive seasonality and visitor-related stress among individual pandas. Notably, the oldest male had a significantly lower T:C ratio than other males. Our results confirm that the level of sex steroids and cortisol can be assayed by panda hair, and the hair cortisol concentrations correlate significantly with that in faeces with one month lag behind (*r* = 0.68, *P* = 0.03). However, the concentrations of hormones detected in saliva are lower than those in faeces by two orders of magnitude, making it difficult to ensure accuracy. By assessing the applicability of hair, faecal and salivary sampling, we can infer their utility in monitoring the reproductive status and acute and chronic stress levels of giant pandas, thereby providing a means to gauge the success of ongoing habitat restoration efforts and to discuss the feasibility of sample collection from wild populations.

## Teaser

This study demonstrates the potential of using hair as a reliable indicator of chronic stress levels in pandas, emphasizing the role of the relationship between sex hormones and cortisol in monitoring reproductive health and stress in wildlife.

## Introduction

The giant panda (*Ailuropoda melanoleuca*) is an iconic flagship species for conservation, both within its native range in China and internationally (Yao *et al.*, 2023). Thanks to concerted conservation efforts, the giant panda has been brought back from the brink of extinction, increasing from only around 1000 pandas living in the wild in the 1980s to around 1864 individuals in 2021, utilizing a habitat area of 2 576 595 ha among six mountain areas (Kang, 2022). The Chinese government established the Giant Panda National Park in 2020 by extending the protected area significantly to 2 713 400 ha (Huang *et al.*, 2020). Nevertheless, giant pandas continue to face threats in the wild from mining operations, road construction, farming practices, human settlement and logging (Kang, 2021). This has resulted in a variety of national habitat restoration initiatives (Wang *et al.*, 2023; Yang *et al.*, 2023). In order to assess and mitigate the impacts of these anthropogenic stressors, it is crucial to monitor the stress levels in wild panda populations (Deng *et al.*, 2014). Cortisol is the predominant glucocorticoid produced by mammals in response to stress (Koren *et al.*, 2012). It is secreted through activation of the hypothalamic–pituitary–adrenal (HPA) axis, and once released, cortisol stimulates the mobilization of energy through glucogenesis while down-regulating other life-sustaining functions that do not immediately contribute to survival, such as immunocompetence, growth or reproduction (Möstl and Palme, 2002; Zenth *et al.*, 2022). Thus, it is vital to develop appropriate non-invasive techniques that can detect allostatic cortisol loads in free-ranging animals (Juster *et al.*, 2010).

Captive pandas have been the subject of various studies examining the effects of stressors (Khan *et al.*, 2022), including stress-related impacts on their reproductive success (Yu *et al.*, 2011; Martin-Wintle *et al.*, 2019). Endocrinological monitoring in blood, urine and faeces of both sexes is well established (Owen *et al.*, 2004; Aitken-Palmer *et al.*, 2012; Cai *et al.*, 2017), but different sample types have different characteristics, and thus vary in their suitability depending on study objectives and logistical circumstances (Murphy, 2002). Blood sampling and analysis provide an accurate, reliable and immediate measure of circulating hormone levels (Arevalo *et al.*, 2015; Binder, 2019), but the phlebotomy process can distress even habituated animals and requires extensive training and often sedation (Soulsbury *et al.*, 2020), which can further affect hormone assay veracity (Meyer *et al.*, 2020). Techniques that can detect hormone levels from faecal, urine and saliva samples are less-/non-invasive, and thus offer particular cross-utility for monitoring how wild pandas may be adversely affected by environmental stressors (Yu *et al.*, 2011). Urinary cortisol concentration typically correlates well with blood levels, but with a time-lag of ca. 12–24 h (Jung *et al.*, 2014). Levels are strongly affected by recent stressful events (such as capture and/or restraint), diurnal rhythms and urine sample water content. Furthermore, it is difficult to predict or control the timing and location of urination, and urine can infiltrate the soil and other environments, making it difficult to obtain fresh, uncontaminated samples from known individuals (Kirkpatrick *et al.*, 1990). Salivary cortisol levels exhibit a strong correlation with concurrent circulating plasma cortisol levels (Peeters *et al.*, 2011; Montgomery *et al.*, 2022). Saliva sampling can thus reflect short-term hormone concentrations in animals, making it suitable for monitoring rapid changes in cortisol levels and enabling timely assessment of an animal’s health status for treatment and management purposes (Kao *et al.*, 2018) as well as reproductive condition for breeding purposes (Kutsukake *et al.*, 2009). However, the sampling procedure poses significant challenges and is primarily applied to domesticated and well-habituated captive animals (Verspeek *et al.*, 2021). In contrast to salivary sampling, collection of faecal samples is easier and does not induce stress. Therefore, this technique has become a common method for hormone monitoring in both captive and wild animals (Terio *et al.*, 1999; Deng *et al.*, 2014; Vega *et al.*, 2020). Nevertheless, in the wild, it can be difficult to match samples to specific individuals, and various factors, such as sample age, food content, the donor’s gut microbiome and weather−/environment-dependent decay of the faecal sample can affect the ability to interpret hormone assay results (Abáigar *et al.*, 2010).

**Table 1:** Information about the sex, age at which faecal sampling collection started and the total number of faecal samples collected from each panda

Number	Subjects	Sex	Age at first sampling	Faecal sample number
1	M1	Male	4	88
2	M2	Male	6	86
3	M3	Male	8	84
4	M4	Male	8	88
5	M5	Male	22	84
6	F1	Female	3	86
7	F2	Female	3	90
8	F3	Female	3	87
9	F4	Female	6	88
10	F5	Female	15	86

Note: This study conducted sampling over a period of 1 year, during which the age of the pandas increased. Therefore, the age displayed in Table 1 is the age at the time of the first sampling, which occurred in April 2022.

Steroid hormones can also be extracted from mammalian hair (Heimbürge *et al.*, 2019). In contrast to the samples mentioned above, hair cortisol concentration provides a measure of hormone deposited into hair keratin over a longer-term period and is thus not influenced by proximate stressors (Faresjo *et al.*, 2023). This offers potential for systemic measurements of cortisol and sex-steroid secretion on a weekly, monthly or even seasonal scale (Raul *et al.*, 2004), with increasingly refined assays offering ever-greater accuracy (Cattet *et al.*, 2017). Hormones in the hair reflect the average hormone concentrations during the past 1–3 months, therefore hair cortisol concentrations can be useful for measuring long-term stress (Abdulateef and Mahwi, 2019; Faresjo *et al.*, 2023). This addresses clinical questions that were previously unanswerable by other sampling techniques (Russell *et al.*, 2012). This method has been used successfully in a wide variety of other species for more than a decade (Kersey and Dehnhard, 2014; Azevedo *et al.*, 2019; Pereira *et al.*, 2022; Fokidis *et al.*, 2023), however, to-date, hormone monitoring from hair samples has not been validated for giant pandas. Pandas typically undergo molting every 6 months, usually around May and October in China (Yang *et al.*, 2022). Therefore, the detection of hormones in their hair can provide guidance for establishing criteria to assess their chronic stress levels.

In this study, we used faecal sex steroid and cortisol concentrations as a reference to investigate if testosterone, estradiol, progesterone and cortisol can be measured in panda hair and saliva samples. We also calculated for each male the ratio of testosterone to cortisol (T:C ratio), which can provide a biomarker of stress (De Luccia, 2016) and exercise performance (Maestu *et al.*, 2002). We use our assessment of the accuracy and suitability of hair sampling to extrapolate applications for monitoring the reproductive condition and stress levels experienced by giant pandas living in the wild, as a means to monitor the success of ongoing habitat restoration efforts (Yang *et al.*, 2023).

## Materials and methods

### Subjects and sampling

We collected hair, faecal samples and saliva samples from 10 giant pandas (5 males, 5 females; see Table 1 for detailed information) housed at Beijing Zoo between April 2022 and March 2023. As described previously (Liu *et al.,* 2017), pandas were kept in adjacent but separate enclosures. Each enclosure consisted of an indoor living area equipped with wooden structures for pandas to climb on, wooden platforms for resting, and either an indoor exercise area with glass walls and a roof to allow for natural sunlight and day-night rhythm or an outdoor exercise area. These exercise areas were equipped with swings, ball toys, trees, wooden structures, hammocks and other similar behavioural enrichment. The pandas were fed fresh, washed and chopped bamboo shoots, culms and leaves as their main food, supplemented with carrots, apples and homemade bread. The feeding of different parts of bamboo to the giant pandas varied across seasons. Different parts of the bamboo exhibit significant variations in fibre content across different seasons (Yan *et al.*, 2023a; Yan *et al.*, 2024b). Ice blocks were provided during the hotter months (May–October) to help pandas to keep cool. During the study period, the activity space of giant pandas remained fixed, with consistent surrounding environmental factors including chemosensory cues and neighbours. In this situation of constant resource availability and population density, the interference of competitive territoriality was avoided.

Between April 2022 and March 2023, zookeepers collected faecal samples every 4 days at 8 a.m., within 20 min of defecation, ensuring the samples were not contaminated by urine or water, to standardize for circadian variation in hormone excretion (Owen *et al*., 2005). However, on some occasions, no fresh faeces were available from certain individuals, and therefore, the total number of samples analyzed from each individual differed slightly over the study period (Table 1). Samples were placed in sterile collection bags, frozen immediately and stored at −40°C until endocrinological analyses were performed in April 2023.

Hair samples were collected from each of these individuals during routine blood sampling in January 2023, with one sample collected from each individual. The hair was obtained by shaving a small area on the anterior forearm, where the hair is black, to expose the skin above the saphenous vein and facilitate phlebotomy (Yang *et al.*, 2015; Wang *et al.*, 2017). Each hair sample weighed 1–2 g and was wrapped in aluminium foil before being frozen and stored at −40°C.

Saliva samples were collected from each individual in January and February 2023 by specially trained caretakers, with one sample collected per individual each month. Pandas were enticed with apples to lean on the railings of their enclosures, and saliva was swiftly collected from their mouths using two cotton swabs (with cotton tips extending one centimeter). These oral swabs were then placed in sterile sealed bags under refrigeration and transported on dry ice to the laboratory at the Department of Ecology, Beijing Normal University for processing.

All of these procedures were approved by the Animal Welfare Committee of Beijing Normal University.

### Treatment of samples and hormone extraction

The processing and endocrinological analyses of faecal samples followed the methodology described in a previous study (Deng *et al.*, 2014), albeit with slight modifications. In brief, we freeze-dried the faecal samples at −60°C for 72 hours using a vacuum freeze dryer (LGJ-10 N, Yaxingyi, China). We then ground and sieved each dried sample and transferred 0.2 g into a 15 ml centrifuge tube (Eppendorf, Hamburg, Germany) and added 5 ml of 80% methanol. This mixture was vortexed at 25 Hz for 20 minutes using a vortex mixer (Scientific Industries, New York, USA). The sample was then centrifuged (Eppendorf, Hamburg, Germany) at 4°C and 1500 rpm for 20 minutes, and the supernatant was transferred into a new collection tube and frozen at −40°C until further analysis. Due to the lack of previous studies on the extraction of hormones from giant panda hair, we adapted established and widely used hair hormone extraction methods used in humans (Kirschbaum *et al.*, 2009), bonobos (*Pan paniscus*) (Verspeek *et al.*, 2021) and rhesus monkeys (*Macaca mulatta*) (Kapoor *et al.*, 2018). In brief, we unwrapped each hair sample and transferred it into a 15 ml centrifuge tube containing 3 ml isopropanol. Each hair sample was washed in a shaker three times at 25 Hz for 3 minutes. After each wash cycle, the isopropanol was discarded and replaced with fresh isopropanol. After the third wash, tubes were opened and left at room temperature for 24 hours until the isopropanol had evaporated. We then placed 50 mg of hair into a crushing tube and added two zirconia beads. The crushing tube was placed into an instrument box, and liquid nitrogen was injected into the box. The instrument box was immediately placed into a GENO2010 sample grinder and vibrated at 25 Hz for 1 minute. After adding 1.5 ml of methanol, the crushing tube was placed into a shaker, vibrating of 2.5 Hz for 24 hours. The zirconia beads were removed, and the tube was centrifuged at 4°C and 1500 rpm for 20 minutes. Finally, 1 ml of the supernatant was transferred to a 15 ml centrifuge tube, which was placed into a vacuum dryer. The cold trap temperature was set to −60°C, and the drying chamber parameters were set to 20°C and 1500 rpm. The samples were dried for 3 hours and then sealed and stored in a −40°C freezer.

The analysis of saliva followed the protocol described by Mohan *et al.* (2020). Cotton swabs enriched with giant panda saliva were placed in a perforated double-layer centrifuge tube and centrifuged at 4000 rpm for 10 minutes. The smaller, interior tube was discarded, and the larger, exterior collection tube containing the collected saliva sample was stored at −40°C, with each sample volume exceeding 1 ml.

### Hormone measurements

Hormone concentrations were assessed following our established method (Deng *et al.*, 2014). All hormones were quantified using radioimmunoassay (RIA) with an XH6080 RIA analyzer (Xi’an Nuclear Instrument Factory, Xian, China). Cortisol was analyzed using the iodine [^125^I] cortisol RIA kit (sensitivity = 2 pg/ml), testosterone with the ^125^I testosterone RIA kit (sensitivity = 0.02 ng/ml), estradiol with the ^125^I estradiol RIA kit (sensitivity ≤ 2 pg/ml) and progesterone with the ^125^I progesterone RIA kit (sensitivity < 2 pg/ml). Each assay kit underwent coefficient of variation (CV) testing, with the cortisol RIA kit having a CV of 4.32%, the testosterone RIA kit with a CV of 7.67%, the estradiol RIA kit with a CV of 9.15% and the progesterone RIA kit with a CV of 5.73%. All assay kits were provided by the Beijing North Institute of Biological Technology in Beijing, China.

### Data analysis

Smoothed curves of faecal cortisol and sex steroids (testosterone, estradiol, progesterone) levels of giant pandas throughout the year were fitted using the Locally Weighted Regression (LWR) method. This method estimates the smoothed curve by fitting a weighted regression around each data point (Leung *et al*., 2004). We performed *t*-tests to test for sex differences in faecal cortisol concentrations between males and females, for inter-individual differences in faecal cortisol, testosterone concentrations in males, progesterone/cortisol ratios, estradiol- and progesterone concentrations in females, as well as for monthly differences in faecal testosterone in males, and estradiol and progesterone concentrations in females. Pearson correlation was used to test for any potential correlation between faecal cortisol and sex hormones including testosterone, progesterone and estradiol concentrations in giant pandas. False discovery rate was controlled using the Benjamini–Hochberg (BH) method, and subsequent analyses were conducted using adjusted *P*-values with a significance criterion of 0.05.

To determine the indicative role of hair cortisol to assess long-term stress in individual giant pandas, we conducted correlation analyses between their hair cortisol concentrations and their average cortisol concentrations in faeces collected 1, 2 and 3 months prior to the hair collection date. We chose these three intervals because previous studies have shown that cortisol concentration in the hair reflect the average concentrations during the past 1–3 months (Abdulateef and Mahwi, 2019; Faresjo *et al.*, 2023). As the hair and faecal cortisol concentration data followed a normal distribution (*P-*value > 0.05; Shapiro–Wilk test), Pearson rank correlations were used. Due to the small sample size of only five individuals for each sex, there was a heightened risk of type II errors, where test statistics are typically less sensitive and may fail to detect true correlations (Knudson and Lindsey, 2013). Therefore, correlation tests were not included in the sex-steroid analyses. Where applicable, hormone concentrations are given as mean ± standard error (SE).

## Results

A total of 867 faecal samples were collected from 10 giant pandas over a period of one year (Table 1). Saliva was collected from each individual twice, once in January and once in February, resulting in 20 saliva samples. In addition, one hair sample was collected from each panda in January.

### Sex steroid patterns in faeces, hair and saliva

#### Patterns in male testosterone

**Figure 1:**
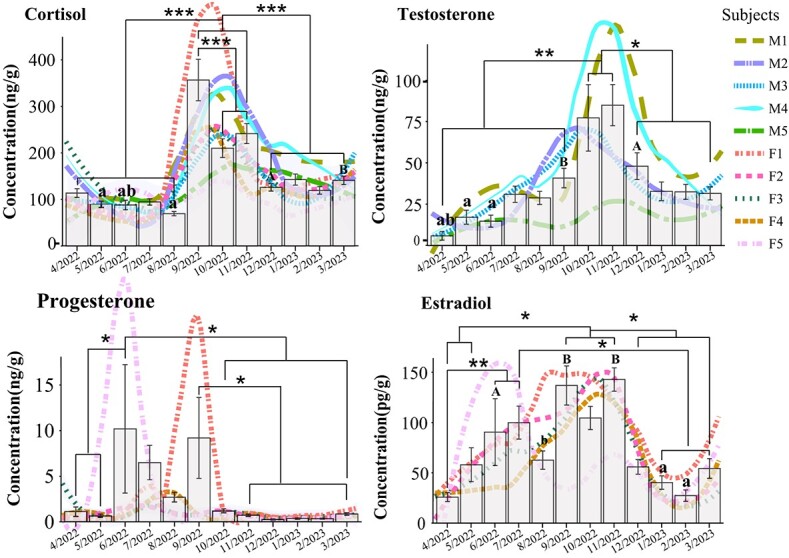
Annual cyclic changes and monthly differences in cortisol, testosterone, progesterone and estradiol as measured in faecal samples in captive giant pandas. The bar graph shows the differences in cortisol, testosterone, progesterone and estradiol concentrations among giant pandas in different months of the year. (^***^ adjusted *P* value < 0.001, ^**^ adjusted *P* value < 0.01, ^*^ adjusted *P* value < 0.05; “A” and “a” as well as “B” and “b” denote *P* value < 0.05; error bars represent standard error). The curve shows the variation among individuals. Data were fitted by locally weighted regression (LWR) method according to month.

**Figure 2:**
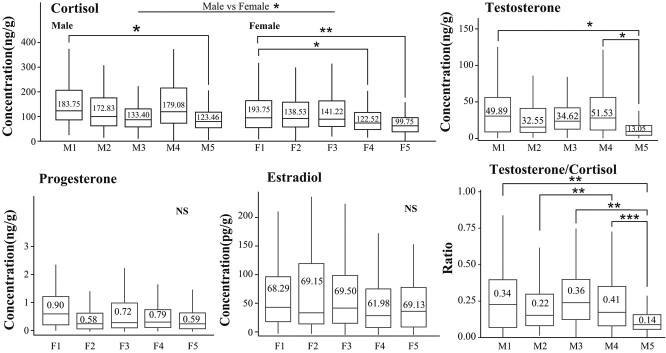
Box-plots of inter-individual differences in faecal cortisol, testosterone, progesterone, estradiol and T:C ratio among captive giant pandas. The horizontal line inside the box represents the median, and numerical values indicate the mean. The statistical significance levels are denoted as follows: ^***^ adjusted *P* value < 0.001, ^**^ adjusted *P* value < 0.01, ^*^ adjusted *P* value < 0.05, NS means *P* > 0.05.

Faecal testosterone in four of the five males were low between April and August, increased during September to November and declined from December to March, but not to concentrations previously measured in April, May and June (Fig. 1). Consequently, concentrations were significantly higher in October and November than they were in any other months (t-test, *P* < 0.05). Throughout the study period, the oldest male had much lower concentrations (13.05 ± 1.40 ng/g) than any of the other males (Fig. 2).

Testosterone concentrations measured in the hair samples collected in January were on average 1.354 ± 0.62 ng/g. Concentrations in saliva collected in January were 0.038 ± 0.023 ng/g and in February 0.18 ± 0.16 ng/g.

#### Patterns in female sex steroids

Female faecal progesterone profiles showed distinct inter-individual differences, one individual (F5) exhibiting a clear peak at the end of May and another individual (F1) at the end of August (Fig. 1). Progesterone concentrations among the other three females did not show significant monthly variation and remained comparatively low throughout the year (0.92 ± 0.11 ng/g) (Fig. 1). There were no significant differences in faecal progesterone concentrations among individuals throughout the year (t-test, *P* > 0.05). In hair, the average progesterone concentration was 0.076 ± 0.029 ng/g (Table 2). Progesterone could not be detected in any of the saliva samples.

**Table 2:** Concentrations of cortisol, testosterone, progesterone and estradiol in giant panda hair samples

Subjects	Cortisol (ng/g)	Testosterone (ng/g)	Progesterone (ng/g)	Estradiol (pg/g)
M1	20.59	2.94		
M2	12.58	0.31		
M3	9.95	0.48		
M4	13.26	2.8		
M5	13.17	0.24		
F1	9.07		0.06	2.1
F2	15.57		0.02	24.01
F3	19.93		0.18	97.55
F4	13.97		0.09	426.12
F5	7.76		0.03	10.95
Mean ± SE	13.59 ± 1.27	1.35 ± 0.62	0.076 ± 0.029	112.15 ± 82.28

The lowest monthly faecal estradiol concentration was in April (25.82 ± 4.47 ng/g), and the highest concentration was observed in November (142.73 ± 11.62 ng/g) (Fig. 1). It is interesting to note that the two females that exhibited clear progesterone peaks reached estradiol peak levels much earlier in the year than the other three females (F5 in mid-June at 44.27 ± 16.06 ng/g and F1 in mid-October at 42.46 ± 11.43 ng/g). Both estradiol and progesterone peaked simultaneously (Fig. 1). Overall, there were no significant inter-individual differences in estradiol concentrations throughout the year (t-test, *P* > 0.05).

The average estradiol concentration in hair in January was 112.15 ± 80.28 pg/g (Table 2), and in saliva, 1.64 ± 0.38 ng/g in January and 1.18 ± 0.61 ng/g in February (N.B., due to the limited amount of saliva collected, estradiol was only detectable in 7 of 10 samples; Table 3).

**Table 3:** Concentrations of cortisol, testosterone, progesterone and estradiol in giant panda salivary samples collected in January and February

Subjects	Cortisol (ng/mL)	Testosterone (ng/mL)	Estradiol (pg/mL)
M1	1.3, 5.16	0.01, 0.81	
M2	1.86, 3.7	0.02, 0.05	
M3	2.45, 3.41	0.02, 0.02	
M4	2.78, 4.21	0.13, 0.03	
M5	2.88, 2.83	0.01, 0.004	
F1	3.43, 2.16		0.9, −
F2	1.58, 0.93		-, 0.19
F3	5.84, 2.86		-, 1.65
F4	1.55, 2.49		2.14, 0.17
F5	1.54, 1.39		1.87, 2.69
Mean ± SE	2.52 ± 0.43, 2.91 ± 0.40	0.038 ± 0.023, 0.18 ± 0.16	1.64 ± 0.38, 1.18 ± 0.61

The monitoring results of saliva samples collected in January and February are separated by a comma. In the table for estradiol detection results, “-” indicates that it was not detected.

### Patterns in cortisol measurements

Overall, faecal cortisol concentrations were lower between April and August (ranging from 69.42 ± 4.62 to 113.66 ± 9.40 ng/g), increased to reach the highest peak in September (381.24 ± 49.75 ng/g), and then returned to pre-peak concentrations (Fig. 1). Thus, across all individuals, monthly cortisol concentrations between September and November were significantly higher than in any other months (t-test, *P* < 0.001) (Fig. 1). Moreover, male cortisol concentrations were significantly higher (159.0 ± 7.1 ng/g, ranging from 121.89 to 183.49 ng/g) compared to females (137.6 ± 8.15 ng/g, ranging from 99.21 to 188.81 ng/g) on annual scale (t-test, *P* = 0.045). In both sexes, the oldest individuals had the lowest and the youngest individuals the highest cortisol concentrations on annual scale (males: *t*-test _M1 vs M5_, *P* = 0.036; females: *t*-test _F1 vs F5_, *P* = 0.003; *t*-test _F1 vs F4_, *P* = 0.044) (Fig. 2).

Average cortisol concentrations across both sexes in hair were 13.59 ng/g ± 1.27 (males: 13.91 ± 1.77; females: 13.26 ± 2.21), and there was no significant difference between the sexes (*P* = 0.82, t-test). Hair cortisol concentrations correlated moderately with the integrated concentrations of faecal cortisol from the previous 1, 2 and 3 months. The strongest correlation was observed with the monthly faecal cortisol concentrations from 1 month prior, reaching significance (*r* = 0.68, *P* = 0.03) (Table 4).

**Table 4:** Correlation between cortisol concentrations in giant panda hair and faecal samples collected 1, 2 or 3 months before the hair was collected

Cortisol	Correlation coefficient	*P* value
Hair and faeces (1 month)	0.68	0.03
Hair and faeces (2 months)	0.38	0.28
Hair and faeces (3 months)	0.37	0.29

Faecal cortisol concentrations represent the average values from 1, 2 and 3 months prior to hair sample collection.

Cortisol was detected in all saliva samples. The average cortisol concentrations across both sexes in January were 2.52 ng/ml ± 0.43 (males: 2.25 ± 0.29; females: 2.79 ± 0.85). There was no significant sexual difference in saliva cortisol levels (*P* > 0.99, Wilcoxon test). In February, the average cortisol concentrations were 2.91 ng/ml ± 0.40 (males: 3.89 ± 0.39; females: 1.97 ± 0.35) with males exhibiting significantly higher cortisol concentrations than females (*P* = 0.072, *t*-test).

### Relationships between faecal cortisol and sex steroids

Testosterone was positively correlated with cortisol concentrations in all five males (*r* = 0.429, *P* < 0.001) (Table 5). In females, there was a moderate correlation of cortisol with estradiol (*r* = 0.400, *P* < 0.001) (Table 5).

**Table 5:** Correlations among cortisol, testosterone, progesterone and estradiol concentrations in giant panda faecal samples

Subjects	Cortisol and testosterone		Cortisol and progesterone		Progesterone and estradiol		Estradiol and cortisol	
	*r*	*P*	*r*	*P*	*r*	*P*	*r*	*P*
M1	0.34	0.00						
M2	0.61	0.00						
M3	0.71	0.00						
M4	0.33	0.00						
M5	0.58	0.00						
All males	0.43	0.00						
F1			0.43	0.00	0.42	0.00	0.41	0.00
F2			−0.02	0.88	0.57	0.00	0.39	0.00
F3			0.40	0.19	0.19	0.08	0.50	0.00
F4			−0.15	0.19	0.21	0.06	0.51	0.00
F5			0.42	0.00	0.91	0.00	0.49	0.00
All females			0.26	0.00	0.60	0.00	0.40	0.00

The Pearson method was used for the analysis, and the *r* value represents the correlation coefficient.

Testosterone to cortisol (T:C) ratio of the oldest male panda (M5) was significantly lower than T:C for M1, M3 and M4 (*t*-test, *P* < 0.01) but no different than the ratio measure for M2 (Fig. 2).

## Discussion

Developing non-invasive tools to monitor giant panda sex steroids and how these interact with the stress-related hormone cortisol is important not only for breeding programs and the welfare of captive giant pandas (Yu *et al.*, 2011) but also as a means to assess the performance of strategies designed to reduce disturbances in wild panda populations (Zhou *et al.*, 2020). In this proof-of-concept study, our analyses established that sex steroids and cortisol could be detected in hair samples collected from captive giant pandas; however, hormone concentrations in hair were approximately an order of magnitude lower than the that detected in faecal samples from these same individuals (except for estradiol). This magnitude difference is likely attributed to the fact that hormones in hair are embedded within a keratin matrix, whereas faecal hormones are mixed into fibrous faecal waste. Salivary concentrations of hormones were again approximately an order of magnitude lower than in hair, and in fact so low that not all hormones could be detected accurately in all samples. Nevertheless, there was a significant correlation between cortisol concentrations measured in faeces and in hair over the period of the preceding 1 month. Many factors may influence the result of this correlation. For example, the rate of hair growth and potential clipping practices at the surface (Abdulateef and Mahwi, 2019; Faresjo *et al.*, 2023). Stressful events may also lead to simultaneous increases in cortisol levels in both hair and faeces (Liu *et al.*, 2006). Moreover, environmental factors such as captivity conditions and social environment may influence stress levels in pandas (Yu *et al.*, 2011), thereby affecting cortisol concentrations in both hair and faeces, and contributing to the observed correlation. Consequently, hair cortisol levels can serve as an indicator for retrospective stress assessment in captive as well as in wild giant pandas.

Cortisol levels in wild animals relate to the stress they experience (e.g. social rank competition) (Bartoš *et al.*, 2010) and influence life-history variation (Crespi *et al.*, 2013). High levels of cortisol secretion, especially if sustained over prolonged periods of time, can result in serious illness (El-Farhan *et al.*, 2017) such as Cushing’s syndrome (Nishiyama *et al.*, 2022). Our 1-year monitoring of faecal hormones in captive giant pandas at Beijing Zoo showed that cortisol levels reached their highest values in late September to early October and their lowest values in late July. Early October is also the peak tourist season at Beijing Zoo, which may cause severe disruption for the giant pandas, where other studies have shown a significant positive correlation between cortisol metabolite levels and the degree of human disturbance and stereotypic behaviour in captivity (Liu *et al.*, 2006; Powell *et al.*, 2006; Deng *et al.*, 2014). Seasonal variation in cortisol levels occurs, however, also in the wild. For example, in brown bears, the cortisol levels tend to be greater in hair samples obtained from late summer and fall than in samples from spring (Cattet *et al.*, 2014).

Male giant pandas are seasonal breeders (Kersey *et al.*, 2010), and so we collected faecal samples across a whole year to detect the male’s seasonal pulse in testosterone production. Interestingly, we found that the testosterone concentrations of giant pandas at Beijing Zoo reached their peak in early October and November, which is in contrast to previous studies on wild giant pandas in the Qinling Mountains (Nie *et al.*, 2012) that showed an increase in testosterone metabolites in February, reaching a peak in March and April, and then returning to baseline levels after the mating season. This difference may be due to the fact that giant pandas at the Beijing Zoo do not participate in breeding, and the Beijing day-night rhythm differs slightly from the Qinling Mountain range.

It is noteworthy that testosterone and cortisol levels in five male giant pandas showed a significant positive correlation. Previous studies have shown that cortisol and testosterone share cholesterol precursors for biosynthesis (Acconcia and Marino, 2018), but while testosterone has a synthetic metabolic effect (Zitzmann and Nieschlag, 2001), cortisol has a catabolic metabolic effect (Brownlee *et al.*, 2005), thus exerting opposing effects (Cumming *et al.*, 1983; Kraemer *et al.*, 2020). Therefore, it is generally believed that high levels of cortisol secretion can disrupt testosterone biosynthesis. For example, high levels of glucocorticoids have a direct inhibitory effect on luteinizing hormone receptors in the interstitial cells of rat testes, leading to reduced testosterone production (Bambino and Hsueh, 1981), a similar effect observed in human study (Cumming *et al.*, 1983). However, numerous studies have also shown a positive correlation between testosterone and cortisol levels under stress conditions (Popma *et al.*, 2007; Mehta and Josephs, 2010; Sigurdsson *et al.*, 2014; Mehta *et al.*, 2015a; Mehta *et al.*, 2015b). When faced with competition and social pressure, cortisol and testosterone concentrations increase simultaneously under the combined action of the HPA and hypothalamic–pituitary-gonadal (HPG) axes, driving behavioural responses to social challenges (Geniole *et al.*, 2017). For example, male Brandt’s voles (*Lasiopodomys brandtii*) show simultaneous increases in cortisol and testosterone levels under high-pressure conditions (Liu *et al.*, 2020). The authors suggest that high-pressure situations increase testosterone concentrations by activating the HPG axis, leading to increased aggressive behaviour in mice. These studies indicate that under stressful conditions, the simultaneous increase in cortisol and testosterone levels leads to a positive correlation between the two. Additionally, this correlation also reflects the reproductive status of wild animals. One study found that captive adult male African (*Loxodonta africana*) and Asian (*Elephas maximus*) elephants showed a positive correlation between cortisol and testosterone concentrations during estrus but not during non-estrus periods (Brown *et al.*, 2007). Another study showed that cortisol levels in reproductively competent adult male wild horses were negatively correlated with testosterone levels, while in non-reproductive males, they were positively correlated (Medill *et al.*, 2023). The male giant pandas in this study were not involved in reproduction and showed no signs of estrus. Therefore, the positive correlation between testosterone and cortisol levels may reflect the reproductive status of these males. This correlation could also serve as a reference for assessing their reproductive potential in the future.

Additionally, our results revealed that testosterone levels and the testosterone to cortisol (T:C) ratio were lowest in elderly male giant pandas. A decrease in the T:C ratio indicates excessive stress (De Luccia, 2016), accompanied by decreased physical performance and body condition (Maestu *et al.*, 2002; Brownlee *et al.*, 2005; Handziski *et al.*, 2006; Castro-Sepulveda *et al.*, 2019; Hough *et al.*, 2021). Muscle strength is crucial for the natural mating behaviour of male pandas, and a decline in muscle function can inhibit their reproductive potential. Decreased levels of male hormones and increased stereotypical behaviour are also important factors inhibiting the reproductive capacity of captive male animals (Diez-Leon *et al.*, 2013). Conversely, individuals with high T:C ratios are likely to occupy higher social ranks (Sherman *et al.*, 2016). Furthermore, testosterone can reverse glucocorticoid-induced muscle atrophy (Zhao *et al.*, 2008). The elderly male giant pandas in this study were 22 years old and no longer involved in reproduction. Generally, giant pandas over 18 years old are considered senile, with decreased activity and reproductive capabilities (Maestu *et al.*, 2002; Castro-Sepulveda *et al.,* 2019; Hough *et al.*, 2021). Therefore, the low T:C ratio in elderly giant pandas is associated with decreased physical and reproductive capabilities, and the T:C ratio provides valuable information for assessing the physiological status and reproductive potential of male giant pandas.

In females, the only source of testosterone production is the adrenal cortex, which is vulnerable to disruption by high cortisol levels (Brownlee *et al.*, 2005). Thus, high T:C ratios are associated with even greater health effects in females, such as atherosclerosis and ischemic heart disease (Lee *et al.*, 2016; Liu *et al.*, 2023). Health and reproductive monitoring programs in giant pandas should therefore consider measuring the T:C ratio also in females.

We also found a moderate positive correlation between cortisol and estradiol concentrations in females, which is consistent with previous studies (Kersey *et al.,* 2011). Corticosteroids can selectively release and induce ovulation of follicle-stimulating hormone (FSH) both *in vitro* and *in vivo* (Brann and Mahesh, 1991). This suggests that while supporting the metabolic demands and physical capabilities of female giant pandas during courtship and mating, cortisol may also promote ovarian and follicular function. Therefore, reproductive status is an important covariate to consider when using cortisol concentrations as an index of stress.

Based on our study, we recommend conducting additional studies to further explore the relationship between hair hormone concentrations and faecal hormones in giant pandas. Firstly, we propose collecting hair samples in May and November to evaluate potential correlations between hair and faecal hormone concentrations over time. This investigation would help determine whether fluctuations in faecal hormone levels are mirrored in hair samples collected in subsequent months. However, considering the timing of molting, it is essential to acknowledge potential confounding factors. Therefore, additional considerations, such as the timing of molting and the age of hair samples, should be taken into account to avoid misinterpretation of results. Furthermore, we suggest conducting a hair marking study to enhance our understanding of hair molting and growth in giant pandas. One approach may involve administering ^13^C and ^15^N labelled glycine, followed by stable-isotope analysis of hair sections. This method would enable researchers to track the growth and turnover of individual hair strands, providing valuable insights into the dynamics of hair hormone concentrations and shedding patterns. Lastly, as giant pandas consume different parts of bamboo with varying fibre compositions across seasons, this can alter the pandas’ gut microbiota and faecal metabolites (Yan *et al.*, 2021; Yan *et al.*, 2023b). Studies in men and boars have shown that different fibre contents can impact testosterone metabolism (Dorgan *et al.*, 1996; Lin *et al.*, 2022). Therefore, the fibre content in bamboo food may influence the metabolism of androgens in giant pandas. Particularly in captive conditions, where giant pandas exhibit poor natural breeding abilities (Yan *et al.*, 2024a), affecting their genetic diversity, further exploration of the influence of bamboo food fibre composition on sex hormone secretion in giant pandas is crucial for addressing the challenges of their reproduction in captivity.

## Conclusion

In this proof-of-concept study, we have successfully extracted cortisol, testosterone, progesterone and estradiol from the hair and faeces of giant pandas, as well as all these hormones except progesterone from saliva samples. This method offers a non-invasive and minimally disruptive approach to monitoring the reproductive status, and assessing acute and chronic stress levels in both captive and wild panda populations. Furthermore, the correlation between testosterone and cortisol in male giant pandas, as well as their ratio, can serve as a reference for assessing their reproductive potential. These findings provide valuable insights into stress assessment and reproductive potential in giant pandas during and after the establishment the Giant Panda National Park.

## Data Availability

The detection data from this study have been presented in the tables and can also be requested from the corresponding author.
